# COVID-19 IMPACT ON HEALTH JOURNEYS FOR VETERANS WITH CHRONIC CONDITIONS

**DOI:** 10.1093/geroni/igad104.3252

**Published:** 2023-12-21

**Authors:** Sheikh Hossain, Kimberly Judon, Matthew Augustine, Sheldon Brown, Kenneth Boockvar, Michael Dolamore, Madeline Villalba, Abigail Baim-Lance

**Affiliations:** James J. Peters VA Medical Center, Bronx, New York, United States; James J. Peters VA Medical Center, Bronx, New York, United States; James J. Peters VA Medical Center, Bronx, New York, United States; James J. Peters VA Medical Center, Bronx, New York, United States; University of Alabama Birmingham, Birmingham, Alabama, United States; VA- Hudson Valley Health Care System, Wappingers Falls, New York, United States; Icahn School of Medicine at Mount Sinai, New York City, New York, United States; James J. Peters VA Medical Center, Bronx, New York, United States

## Abstract

The majority of Veterans receiving care in the Veterans Administration (VA) are over 65 years, and many have multiple chronic conditions and functional limitations. In this mixed methods pilot study, we sought to understand how these Veterans experienced the pandemic and associated VA health system changes across two contrasting (rural/urban) VA New York medical settings. From December 2021-January 2023, we conducted semi-structured interviews including co-created journey maps and extracted participants’ demographic and clinical characteristics and outpatient encounters. Journey maps visualized health and related episodes and associated pain and bright spots calibrated by intensity. 40 Veterans participated. An intense COVID-19 reaction emerged in 25% (10/39) of participant journeys. A majority (80%; 8/10) had a mental health (MH) diagnosis, compared to 55% (21/39) overall. A journey typically began with feeling shocked, afraid, or in extreme distress; subsequently there was divergence with some expressing feeling better and others describing lingering negative effects on daily routines, views of a changed world, and persistent VA service impacts. Some reported new perceptions of vulnerability due to age and decreased well-being. Participants in this group used on average more outpatient care before and during the pandemic than other participants and transitioned to greater consumption of virtual care, reflecting utilization trends across participants with mental health diagnoses. The group had mixed feelings about the benefits of virtual care. Ongoing challenges with COVID-19 may indicate the potential need for additional well-designed supportive services and environments particularly sensitive to the long-term impact of COVID-19.

